# Development of a prediction model for pulmonary nodules using circulating tumor cells combined with the uAI platform

**DOI:** 10.3389/fonc.2025.1594499

**Published:** 2025-07-23

**Authors:** Dahu Ren, Shuangqing Chen, Shicheng Liu, Xiaopeng Zhang, Wenfei Xue, Qingtao Zhao, Guochen Duan

**Affiliations:** ^1^ Department of Thoracic Surgery, Hebei General Hospital, Shijiazhuang, Hebei, China; ^2^ Graduate School, Hebei Medical University, Shijiazhuang, Hebei, China

**Keywords:** pulmonary nodule, artificial intelligence, circulating tumor cells, early lung adenocarcinoma, prediction mode

## Abstract

**Objective:**

To explore the clinical application value of combining circulating tumor cell (CTC) detection with the artificial intelligence imaging software “uAI platform” in predicting the pathological nature of pulmonary nodules (PN). Develop a joint diagnostic system based on the uAI platform and quantitative detection of CTCs, enable simultaneous classification of pulmonary nodules as benign or malignant and assess the degree of infiltration.

**Methods:**

A total of 76 patients with pulmonary nodules undergoing surgical treatment were enrolled. Preoperatively, three-dimensional nodule risk stratification (low、medium、high risk) was performed using the uAI platform, and CTC high-throughput detection was conducted. Key indicators were selected through multi-group comparisons (Benign、Malignant、Invasive subgroups) and logistic regression analysis. A multi-dimensional nomogram model was constructed, and its clinical utility was evaluated using ROC curves and clinical decision curves.

**Results:**

Comparison between benign and malignant pulmonary nodule groups revealed significant differences in the risk stratification of the uAI platform (proportion of high-risk: 75.61% *vs* 34.29%) and in the median value of CTC quantitative detection (P<0.001). Multivariate logistic regression analysis demonstrated that high-risk classification by uAI and CTC quantitative detection were independent predictors of malignancy in pulmonary nodules (P<0.05). The nomogram model constructed based on these factors exhibited excellent discrimination, and its combined diagnostic performance was significantly better than that of single indicators (AUC=0.805 *vs* uAI 0.730/CTC 0.743).

**Conclusion:**

The combined uAI-CTC model breaks through the limitations of single-dimension diagnosis, enabling risk stratification of malignant pulmonary nodules and quantitative assessment of infiltration, providing evidence-based support for clinical treatment strategies.

## 1 Introduction

Lung cancer remains a significant global public health threat, with the latest epidemiological data from 2024 indicating that its mortality rate continues to rank first among malignant tumors (1). In China, the reported 5-year survival rate for lung cancer is 28.7, which remains relatively low (2, 3). However, the prognosis of lung cancer varies considerably depending on its stage, with the 5-year survival rate reaching up to 92% for stage I lung cancer (4). Nevertheless, the delayed diagnosis of pulmonary nodules (PNs) due to the absence of typical symptoms in their early stages often leads to patients being diagnosed at advanced stages, highlighting the urgent clinical need for the development of precise early screening techniques.

In the field of differentiating benign and malignant PNs, significant advancements have been made in multimodal diagnostic techniques. With the adoption of low-dose computed tomography (LDCT) as the primary screening modality, a 20% reduction in mortality has been achieved among high-risk populations. However, LDCT screening is prone to false-positive diagnoses or underdiagnosis, the baseline false-positive rate was reported as 24% in the National Lung Screening Trial (NLST) (5). Nevertheless, with advancements in imaging technology and follow-up strategies, the postoperative pathological benign rate has progressively declined from 14.5% to 6.2% (6). Radiologists traditionally identify PNs through manual interpretation of chest CT scans—a process that is time-consuming, labor-intensive, and susceptible to diagnostic variability.

In this context, the “uAI platform,” a PNs intelligent diagnostic system developed by Shanghai United Imaging Company, has overcome the limitations of traditional computer-aided diagnosis (CAD). This system is based on a supervised deep transfer learning (SDTL) framework trained on high-quality annotated CT images. It achieves a diagnostic AUC of 91.8% for pulmonary nodules ≤3 cm in size, improves model specificity by 12.3% (7). The uAI platform enables highly sensitive detection of PNs, providing quantitative analysis of multidimensional parameters including PNs size, volume, and composition. This system facilitates comprehensive and precise evaluation of lesion characteristics while offering intelligent prediction of PNs benign/malignant status.

Circulating tumor cells (CTCs) are tumor cells that detach from the primary or metastatic lesions and enter the bloodstream or lymphatic system. These cells have potential value in the diagnosis and monitoring of malignant tumor progression, particularly exhibiting unique advantages in the early diagnosis of lung adenocarcinoma. Studies have already utilized CTCs for the diagnosis of lung adenocarcinoma and lung cancer subtyping (8, 9). However, no research has described their association with the degree of lung cancer invasion. Additionally, the techniques for capturing and isolating CTCs are diverse, and the sensitivity of detection is often limited by the volume of blood samples, such as the use of EpCAM for positive or negative enrichment (10, 11). The GILUPI CellCollector, an *in-vivo* examination technology equipped with a fully automated operating system, enables the counting of CTCs and the detection of tumor-specific protein subtypes, significantly improving the efficiency and accuracy of detection (12).

This study innovatively integrates the PNs risk stratification of the uAI platform with quantitative detection of CTCs, constructing a PNs diagnostic model. It is the first to confirm the synergistic effect of the combined strategy of artificial intelligence and liquid biopsy on the stratification of malignant risk in PNs, providing a practical solution for advancing the early diagnosis and treatment of lung adenocarcinoma.

## 2 Study cohort and methods

### 2.1 Study cohort

This study included patients with PN who were treated at our hospital from January 2018 to July 2023. The inclusion criteria were as follows: 1. Patients who underwent radical surgical treatment for complete resection of PN; 2. The patient’s most recent preoperative CT was analyzed by the uAI platform. PNs have a direct range of 8 mm to 30 mm, including part-solid nodules and solid nodules; 3. The postoperative pathological diagnosis was clear, and the type was LUAD; 4. Complete clinical information of the patient was available; 5. No history of lung cancer or other malignancies in the past 5 years; 6. No clinical symptoms at the time of consultation. The exclusion criteria were: 1. Preoperative CT images of patients could not be recognized and analyzed by artificial intelligence software; 2. History of other pulmonary diseases; 3. Pathologically confirmed metastatic tumors or other types of lung cancer such as squamous cell carcinoma and small cell lung cancer; 4. Patients with distant metastasis; 5. Patient has not undergone surgery or identified pathology by other means (e.g., needle biopsy). After screening, a total of 76 patients were included in this study.

This is a retrospective study conducted at Hebei Provincial People’s Hospital. We obtained approval from the Hospital’s Ethics Committee, waiving the need for informed consent from patients (Ethics No.2023125). We ensured the confidentiality of patient information, and all procedures were in accordance with the Declaration of Helsinki.

Comprehensive clinical data were collected from the patients, including gender, age, family history, smoking history, and tumor markers [such as carcinoembryonic antigen (CEA), squamous cell carcinoma antigen (SCC), cytokeratin 19 fragment (CYFRA21-1), and neuron-specific enolase (NSE)].

### 2.2 Methods

#### 2.2.1 Detection of CTCs *in vivo* using CellCollector

**Figure 1:**
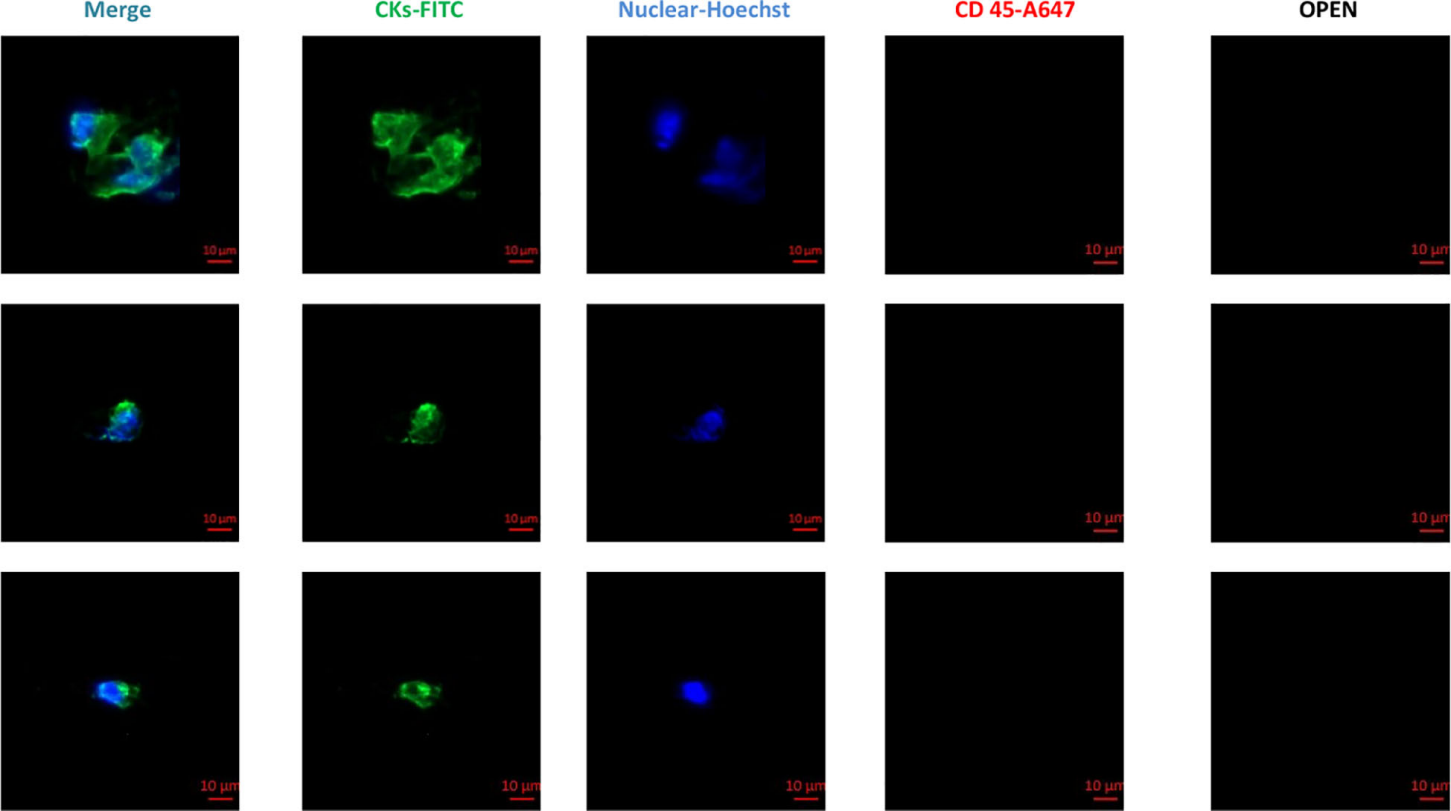
Circulating tumor cell test report: Determination of tumor cells by CK7/19/panCK (green channel). Hoechst was used for nuclear counterstaining (blue channel), and white cells were determined by CD45 staining (red channel). OPEN indicates a reserved blank channel. Scale bar: 10 μm.

For the detection of CTCs in peripheral blood, the CellCollector system was employed. This system utilizes a medically graded stainless steel wire probe as its core component, with the functional domain coated with EpCAM antibodies and hydrogel. The probe was inserted into the elbow vein via a 20G intravenous catheter, ensuring that the functional segment was fully exposed to blood flow for 30 minutes. This allowed for the capture of CTCs via specific EpCAM-antibody binding. Upon completion of sampling, the probe was processed according to a standard staining protocol. As controls, NK92 cells (negative control) and SK-BR-3 cells (positive control) were simultaneously set up. The staining antibodies included CD45 (EXBIO, Clone MEM-28-Alexa Fluor 647) and Cytokeratin CK7/19/panCK antibody (EXBIO Praha, Clone A53-B/A2-Alexa Fluor 488) (**Figure 1**).

#### 2.2.2 Risk stratification of patient PN using the uAI platform

**Figure 2:**
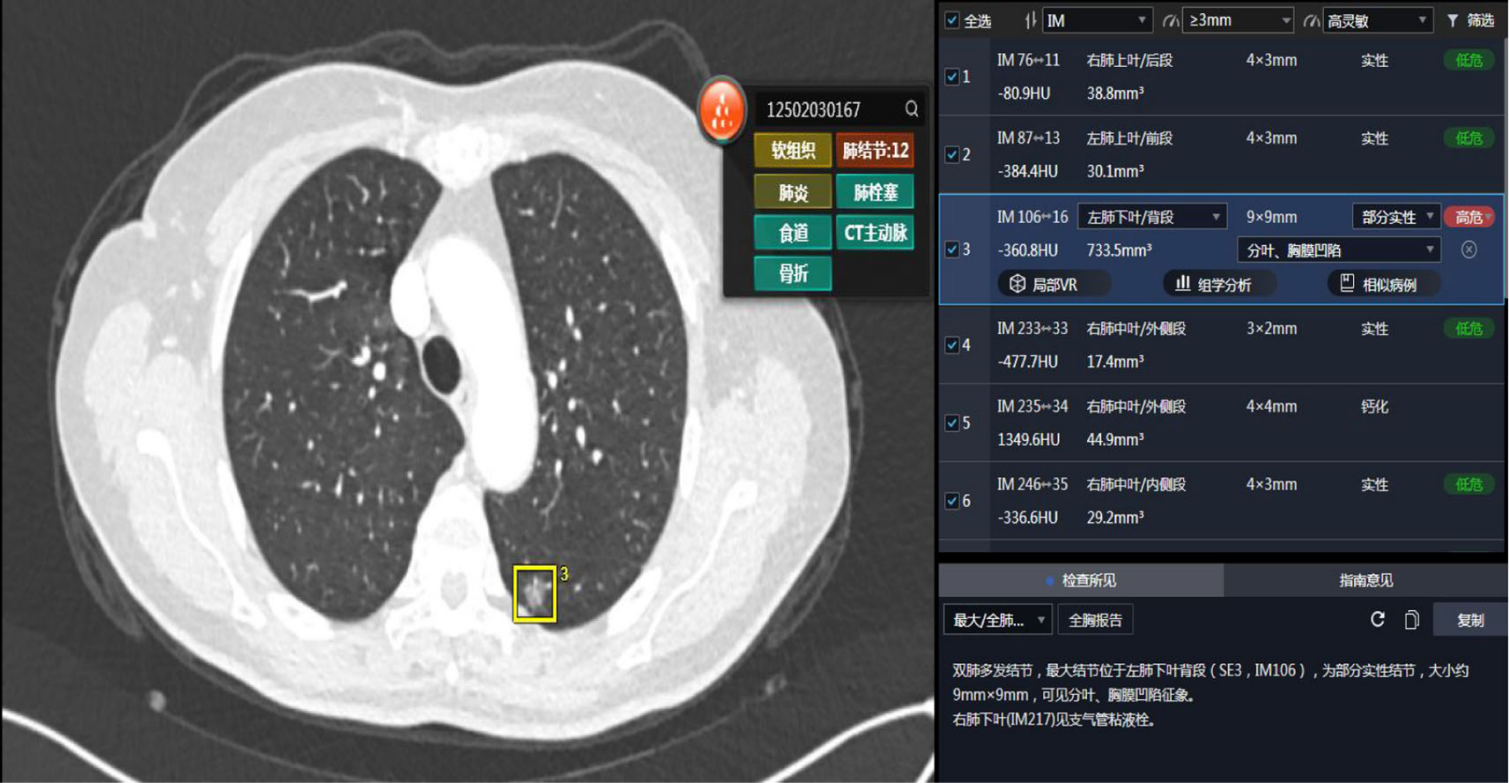
The uAI platform detects PN on thoracic computed tomography (CT) images and conducts artificial intelligence-driven risk stratification of the identified lesions.

The Siemens Somatom Definition Flash system (Siemens Healthineers, Erlangen, Germany) was utilized for CT imaging with a collimation width of 80 mm (128 × 0.625 mm) and a slice thickness of 1 mm. Patients were instructed to hold their breath after inhalation during the scanning process, which covered the range from the superior margin of the thoracic inlet to the adrenal gland level. The complete chest CT images of the patients were uploaded to the uAI platform, where the SDTL was employed to quantitatively analyze multi-dimensional information such as nodule size, volume, density, and composition. This facilitated precise risk stratification of lung nodules into low, medium, high, and very high-risk categories, providing a basis for comprehensive and accurate lesion analysis (**Figure 2**).

### 2.3 Statistical methods

This study used the t-test and chi-square test to accurately describe the association between clinical features and benign and malignant tumors. The “pROC” package in R was utilized to generate ROC curves and calculate the area under the curve (AUC) to evaluate the accuracy of the model. The “rms” package was used to perform univariate and multivariate logistic regression analyses on patients, establish prediction models, and construct nomograms to guide clinical decision-making. GraphPad Prism was employed to analyze the differences in CTC counts among different infiltration groups. The version of R used was 4.3.0, and the version of GraphPad software was 9.4.1.

## 3 Results

### 3.1 Clinical characteristics of subjects

A retrospective analysis was conducted on 76 patients with PN who underwent surgical treatment in our department. **Table 1** describes the clinicopathological characteristics of the study subjects. The study evaluated 43 (56.6%) female and 33 (43.4%) male participants, with a mean age of 59 ± 8 years. Postoperative pathological diagnosis revealed that 35 (46.1%) cases of PN were benign lesions, while 41 (53.9%) cases were lung adenocarcinoma (20 (48.8%) cases of microinvasion and 21 (51.2%) cases of invasion). The uAI platform, through deep learning to quantify nodule features, identified 10 (13.1%) low-risk patients, 23 (30.3%) medium-risk patients, and 43 (56.6%) high-risk patients.

**Table 1:** Association of clinicopathological characteristics in 76 PN patients.

N		Benign	LUAD	P-value
		35	41	
Gender (%)	Female	17 (48.57)	26 (63.41)	0.285
	male	18 (51.43)	15 (36.59)	
Age [mean (SD)]		56 (8)	59 (8)	0.275
Family History (%)	No	29 (82.86)	35 (85.37)	1.000
	Yes	6 (17.14)	6 (14.63)	
Smoking (%)	No	26 (74.29)	23 (56.10)	0.158
	Yes	9 (25.71)	18 (43.90)	
CEA [median (IQR)]		1.87 [1.35, 2.68]	2.52 [1.42, 3.69]	0.216
NSE [median (IQR)]		10.53 [9.29, 14.22]	12.13 [10.73, 13.53]	0.193
CYFRA21-1 [median (IQR)]		1.81 [1.44, 2.67]	1.84 [1.33, 2.35]	0.431
SCC [median (IQR)]		0.97 [0.75, 1.36]	0.98 [0.78, 1.35]	0.790
CTC [median (IQR)]		0.00 [0.00, 1.00]	1.00 [1.00, 2.00]	0.0001
Position (%)	Left	18 (51.43)	12 (29.27)	0.082
	Right	17 (48.57)	29 (70.73)	
Risk (%)	Low	9 (25.71)	1 (2.44)	0.0004
	Medium	14 (40.00)	9 (21.95)	
	High	12 (34.29)	31 (75.61)	

LUAD, Lung Adenocarcinoma; CEA, Carcinoembryonic Antigen; NSE, Neuron-Specific Enolase; CYFRA21-1, Cytokeratin 19 Fragment; SCC, Squamous Cell Carcinoma Antigen; CTC, Circulating Tumor Cell.

### 3.2 Quantitative detection of CTCs

**Figure 3:**
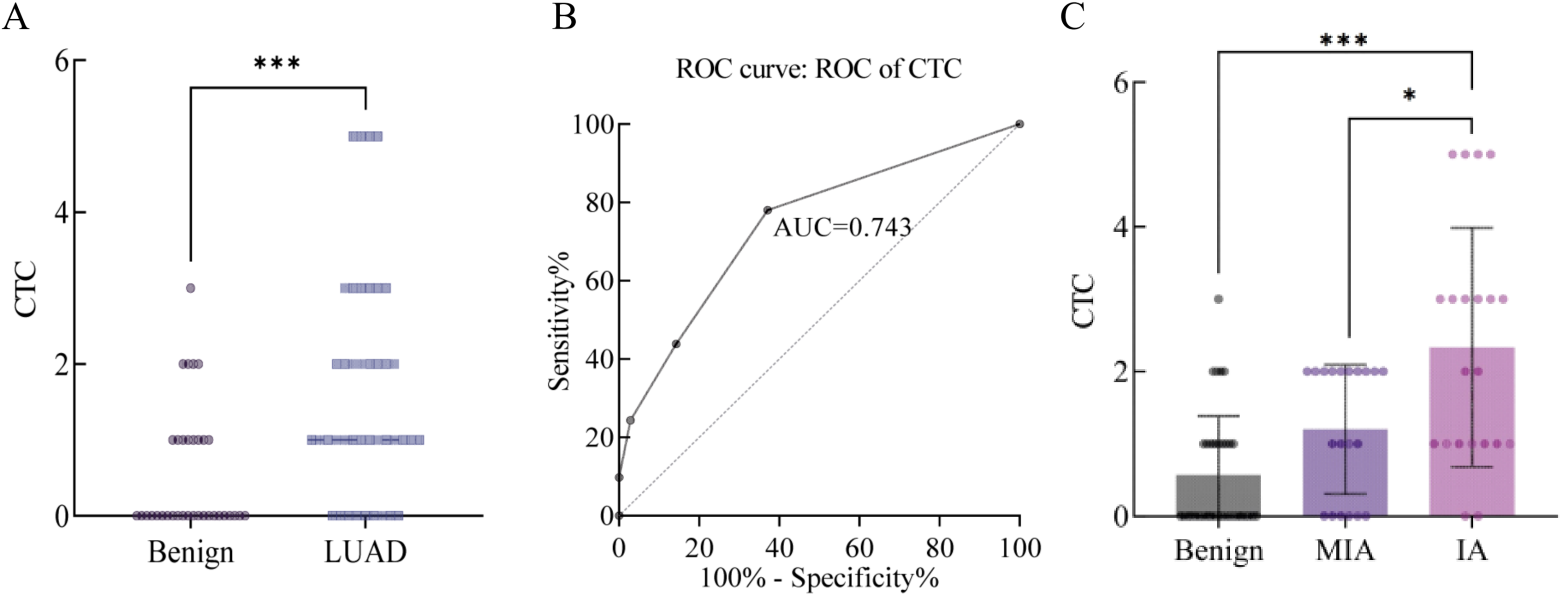
**(A)** Comparative analysis of CTC quantitative detection between benign and malignant groups. **(B)** ROC curve of CTC in differentiating benign and malignant pulmonary nodules, with an area under the curve (AUC) of 74.3%. **(C)** Differential analysis of CTC quantification across varying degrees of invasiveness. (*P<0.05, ***P<0.005).

A differential analysis was conducted on the CTC counts between the benign and malignant groups, revealing a significant difference between the two groups (P<0.001) (**Figure 3A**). The diagnostic performance of CTC counts in PN diagnosis was evaluated using the ROC curve, with an AUC of 74.3% (**Figure 3B**). Further analysis of CTC counts among different degrees of infiltration showed a difference between microinvasion and invasion (P<0.05) (**Figure 3C**).

### 3.3 Risk stratification using the uAI platform

**Figure 4:**
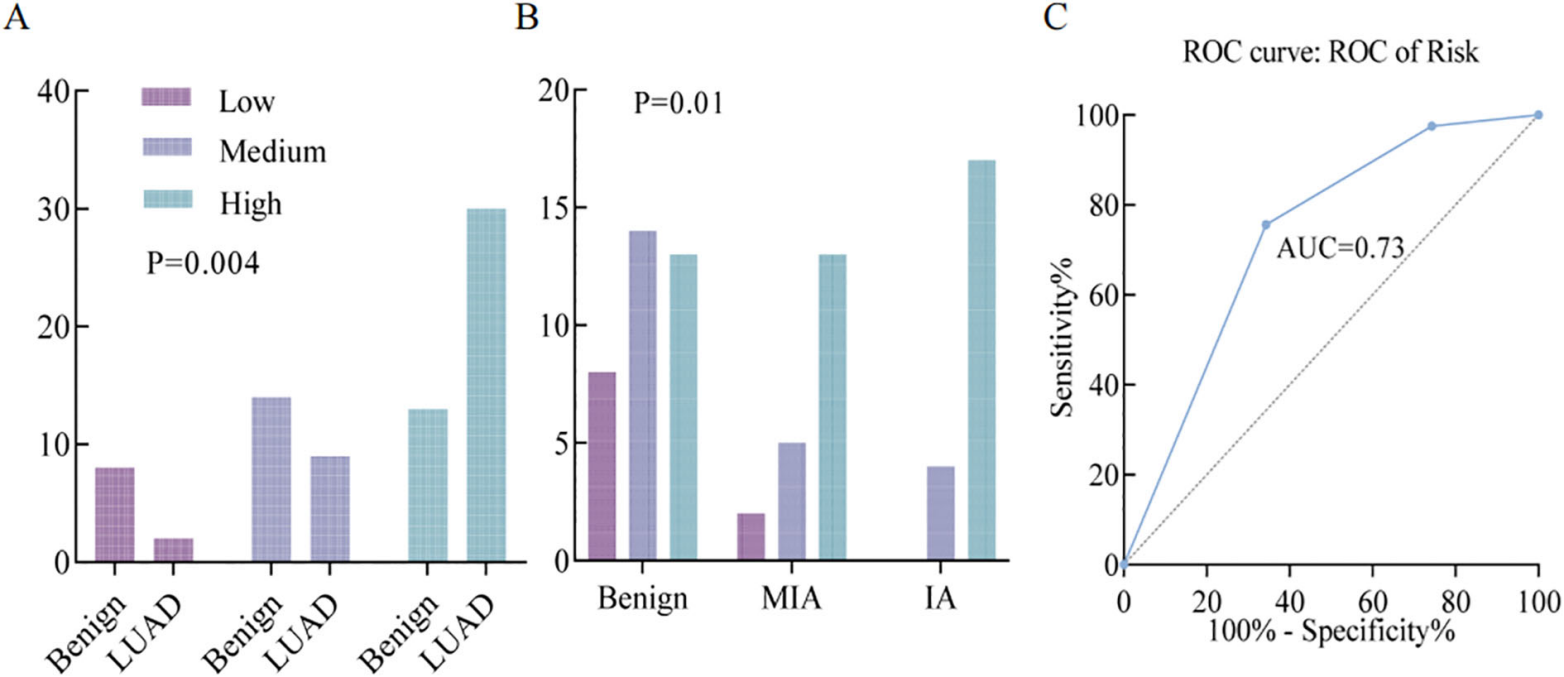
**(A)** Comparative analysis of uAI platform-based risk stratification between benign and malignant groups (*P* = 0.004). **(B)** Differential analysis of uAI platform-derived risk stratification across varying degrees of tumor invasiveness (P = 0.01). **(C)** ROC curve of uAI platform-based risk stratification in differentiating benign and malignant pulmonary nodules, with an AUC of 73.0%.

The results showed that there were significant differences between benign and malignant groups using the uAI platform (P=0.04, **Figure 4A**). The diagnostic performance of uAI Platform count in PN diagnosis was evaluated using the ROC curve, yielding an AUC of 73.0% (**Figure 4C**). Further analysis of different degrees of infiltration showed a significant difference between microinvasion and invasion (P=0.01, **Figure 4B**). These results suggest that risk stratification using the uAI platform is an effective predictive indicator of PN pathological properties. Clinically, the AI software from Shanghai United Imaging Company, has been utilized to assist our department’s physicians in PN diagnosis and treatment.

### 3.4 Logistic regression analysis

Logistic regression analysis was conducted incorporating patients’ clinical information, CTC count, and uAI platform risk stratification. Predictors with P<0.1 in univariate analysis, including nodule location, CTC count, and uAI platform risk stratification, were included in the multivariate analysis. The results revealed that CTC count (OR 2.12, 95%CI 1.16-3.87, P=0.015) and uAI platform risk stratification (OR 11.15, 95%CI 1.18-105.47, P=0.035) served as independent predictors for benign and malignant PN diagnosis (**Table 2**). These findings are consistent with our previous differential analysis.

**Table 2:** Univariate and multivariate logistic regression analysis.

Characteristics	Univariate logistic analysis			Multivariate logistic analysis		
	OR	95%CI	P-value	OR	95%CI	P-value
Age	1.03	0.98-1.09	0.272	NA	NA	NA
CEA	1.17	0.86-1.59	0.327	NA	NA	NA
CTC	2.5	1.45-4.33	0.001	2.12	1.16-3.87	0.015
CYFRA21-1	1.01	0.77-1.33	0.929	NA	NA	NA
Gender	0.54	0.22-1.36	0.195	NA	NA	NA
Family History	0.83	0.24-2.85	0.765	NA	NA	NA
NSE	1.1	0.92-1.31	0.293	NA	NA	NA
Position	2.56	1-6.58	0.051	2.42	0.79-7.42	0.12
Risk						
Medium *vs* Low Risk	5.79	0.62-53.76	0.123	2.64	0.25-27.39	0.42
High *vs* Low Risk	23.25	2.65-203.77	0.004	11.15	1.18-105.47	0.035
SCC	0.71	0.34-1.48	0.362	NA	NA	NA
Smoking	2.26	0.85-6.01	0.102	NA	NA	NA

LUAD, Lung Adenocarcinoma; CEA, Carcinoembryonic Antigen; NSE, Neuron-Specific Enolase; CYFRA21-1, Cytokeratin 19 Fragment; SCC, Squamous Cell Carcinoma Antigen; CTC, Circulating Tumor Cell.

### 3.5 Development of the nomogram

**Figure 5:**
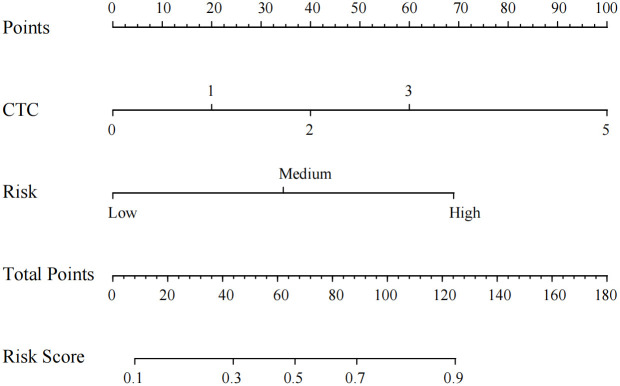
Nomogram prediction model for differentiating benign and malignant pulmonary nodules.

Multi-factor Logistic regression analysis identified CTC count and uAI platform risk stratification as independent predictors of PN status. Based on logistic regression analysis, a nomogram was developed to predict PN characteristics (**Figure 5**).

**Figure 6:**
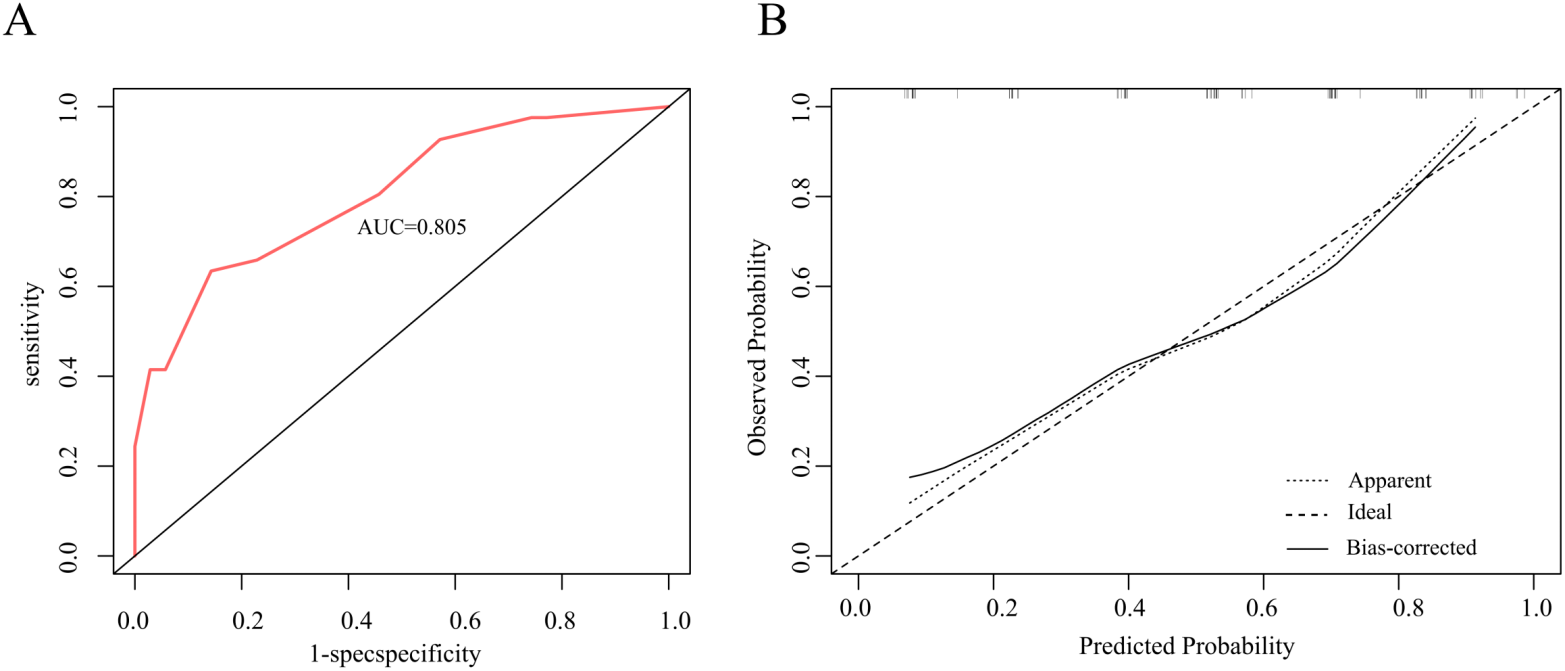
**(A)** ROC curve of Nomogram prediction model, with an AUC of 80.5%. **(B)** The calibration curves Nomogram prediction model.

**Figure 7:**
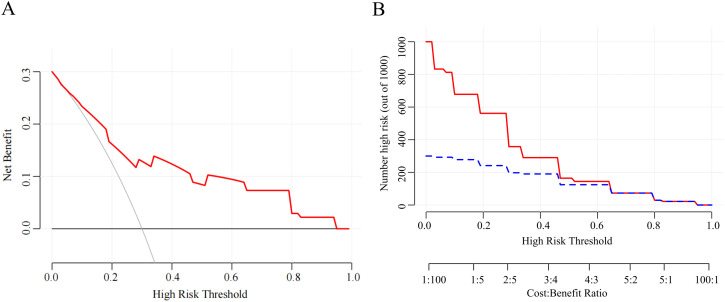
**(A)** Decision curve analysis for the nomogram. The Y-axis shows the net benefit. The X-axis shows the corresponding risk threshold. **(B)** Clinical impact curve for the risk model. Of 1000 patients, the red solid line shows the total number who would be deemed at high risk for each risk threshold. The blue dashed line shows how many of those would be true positives cases.

**Figure 8:**
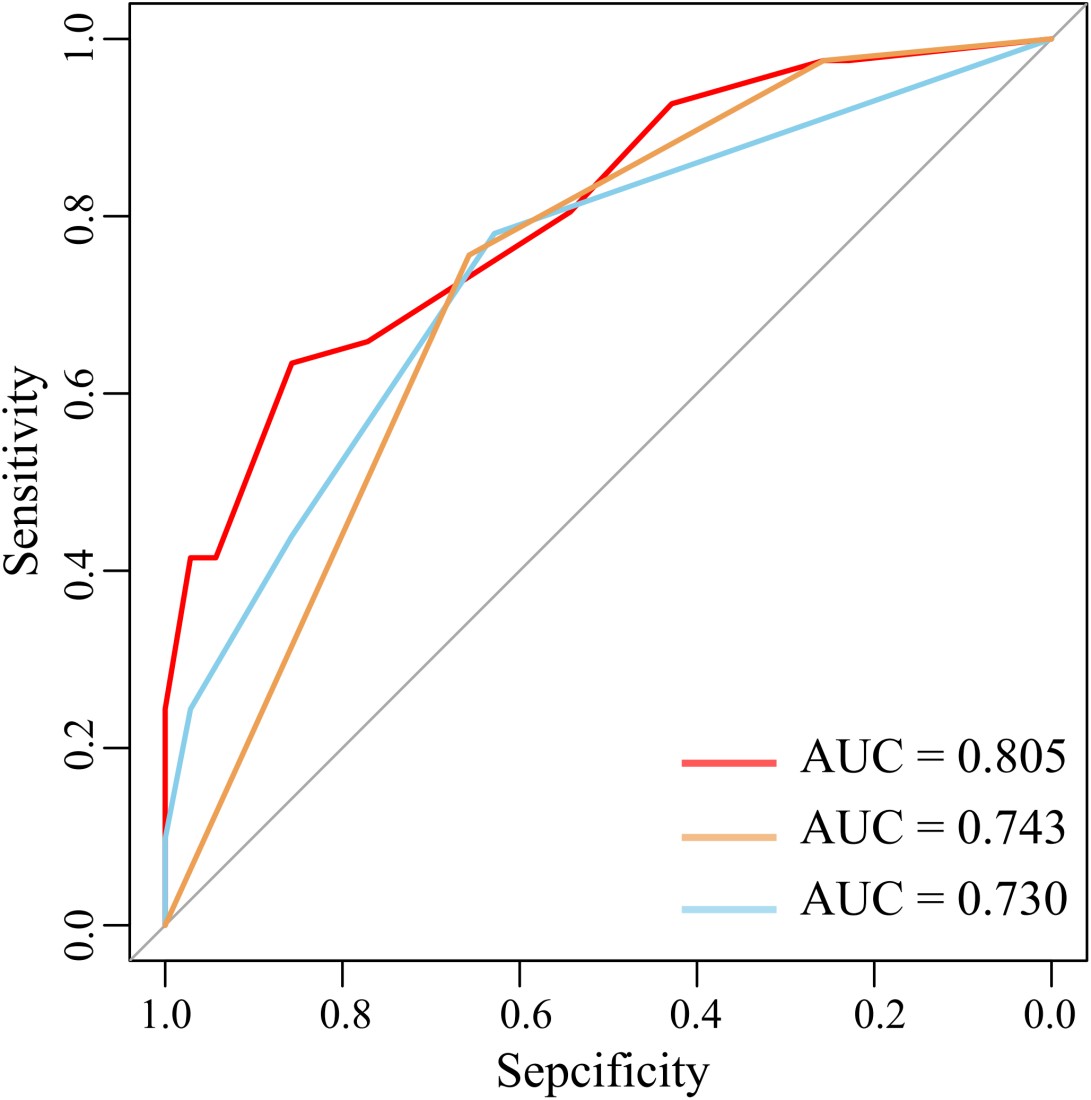
AUC comparison between different groups (Red: Nomogram; Yellow: uAI platform; Blue: CTC).

The nomogram was built using the “rms” package in R, and its accuracy was verified through ROC curves and calibration curves. Decision Curve Analysis (DCA) and Clinical Impact Curve (CIC) analysis were employed to evaluate clinical utility. The constructed nomogram demonstrated excellent discrimination, with an AUC of 80.5% (**Figure 6A**). Internal validation was conducted using 1000 bootstrap replications, confirming the good stability of the nomogram model (**Figure 6B**). The study further assessed the model’s accuracy through 200 iterations of 10-fold cross-validation, achieving an average AUC of 94%, indicating the model’s superiority. Corresponding DCA and CIC analyses (**Figure 7**) revealed that the nomogram provided a superior overall net benefit within the practical range, highlighting the model’s significant predictive value. For the nomogram, the AUC value was 0.805, and the diagnostic performance was higher than that of the uAI platform of 0.743 and CTC of 0.730 (**Figure 8**). However, the AUC difference between different groups was not statistically significant when using bootstrap (Nomogram model/uAI platform P=0.102; Nomogram model/CTC P = 0.058), which may be related to the lower number of patients included in the study.

## 4 Discussion

In recent years, the incidence and mortality rates of lung malignancies have ranked first globally. Studies indicate that the recurrence rate of early-stage lung cancer is only 10% five years after surgery, highlighting the importance of early detection and surgical intervention in the treatment of lung cancer (13). Early-stage lung cancer does not exhibit specific clinical manifestations, and its initial presentation may merely be an isolated pulmonary nodule. Based on the solid components within the nodule, isolated nodules can be categorized into solid nodules and subsolid nodules. Solid nodules refer to those where the nodule density is sufficient to obscure the small bronchi and blood vessels within, while subsolid nodules are characterized by unclear or indefinite boundaries, and the nodule density does not obscure the small bronchi and blood vessels passing through it (14, 15).

Studies have demonstrated that compared to non-small cell lung cancer (NSCLC) presenting as subsolid nodules on imaging, pulmonary lesions manifesting as pure solid nodules exhibit higher rates of lymph node metastasis, greater malignant potential, and poorer prognosis (16, 17). However, the radiological characteristics of pulmonary nodules (PNs) with diameters ≤1 cm are often inconspicuous, leading to interobserver variability in interpretation due to differences in clinical experience (18, 19). In recent years, numerous studies have explored the clinical application of AI-based radiological models for early lung cancer prediction, providing valuable assistance in characterizing PNs. Catelli et al. utilized high-resolution computed tomography (HRCT) features (e.g., spiculation, size, and density) to differentiate benign from malignant nodules, offering clinicians a clearer reference for determining the nature of PNs and formulating surgical strategies (20). Pan et al. proposed an innovative approach by integrating binary and ternary classification models with a pruning decision strategy to resolve classification conflicts, thereby improving the accuracy of predicting the invasiveness of lung adenocarcinoma and demonstrating its potential for CT-based risk stratification in lung cancer (21). Nevertheless, CT radiological characteristics alone cannot fully distinguish benign from malignant PNs. Previous studies have indicated that combining radiological features with additional and more sensitive molecular markers can enhance the diagnostic positivity rate for lung cancer (22–24). Therefore, in this study, we employed uAI platform and CTC assisted analysis to uniformly assess the malignancy risk of patients’ PNs.

Relevant studies have indicated that certain tumor markers in serum can also aid in the diagnosis of lung cancer. However, traditional tumor markers such as CEA, NSE, and CA199 demonstrate limited specificity and sensitivity in the early diagnosis of lung cancer (24–27). In recent years, the emergence of liquid biopsy techniques, represented by CTCs, has provided a novel approach for the early diagnosis of lung cancer. CTCs refer to tumor cells that detach from primary or metastatic lesions and enter the bloodstream. The number of CTCs in peripheral blood is extremely low, and specific CTC markers are crucial for improving CTC detection rates. Folate receptor positivity, as a specific marker for CTCs, exhibits high sensitivity and specificity and is highly expressed in tumor cells. The folate receptor is minimally expressed in the cells of the fallopian tube, renal tubules, alveolar walls, choroid, and uterus, and is not expressed in blood cells. Its expression in lung cancer cells exceeds 78%. Furthermore, the folate receptor can identify active CTCs, unaffected by the transition from epithelial to mesenchymal cells. In this study, the CellCollector (GILUPI CellCollector, GILUPI) was used for *in-vivo* detection of peripheral CTCs before surgery. CTC assessment analysis were performed using immunofluorescence staining. Additionally, CTCs captured by the CellCollector sampling probe were isolated for whole-genome amplification and quality assessment. In 2010, the American Joint Committee on Cancer (AJCC) Cancer Staging Manual proposed that CTCs could serve as a new indicator to assist pathologists in pathological staging. Clinically, CTCs have begun to be used to better develop treatment plans for lung cancer patients (28–30). Previous attempts have been made to combine CTC assessment with established screening methods, focusing on improving specificity by integrating low-dose CT screening programs with subsequent CTC evaluations. CTC enumeration assessments were conducted on patients with identified “ground-glass” lung nodules and healthy controls. CTCs were only present in the blood of some patients with nodules. Based on subsequent molecular analysis, these CTCs were found to have a “malignant tendency” (31). However, the study did not establish a model to predict the pathological nature of nodules and lacked a unified set of diagnosis codes. We retrospectively evaluated the diagnostic efficacy of combining liquid biopsy with artificial intelligence in 76 patients suspected of having lung cancer. The results demonstrated the superior predictive performance of this forecasting model.

This study has several limitations. Firstly, the pulmonary nodules detected in this study were exclusively focused on lung adenocarcinoma; future research should incorporate pathological data from other types of malignant nodules. Second, due to the retrospective nature of this study and the relatively small sample size collected, prospective validation of the hypotheses was unattainable. Further large-scale, multicenter studies are warranted for conclusive verification.

## 5 Conclusion

This study developed a pulmonary nodule prediction model integrating uAI platform and CTCs, demonstrating robust accuracy, stability, and clinical applicability through internal validation. In the future, we will verify it through large-sample, multi-center studies.

## Data Availability

The raw data supporting the conclusions of this article will be made available by the authors, without undue reservation.
